# Association between intraarticular cytokine levels and clinical parameters of osteochondritis dissecans in the ankle

**DOI:** 10.1186/1471-2474-15-169

**Published:** 2014-05-22

**Authors:** Hagen Schmal, Ingo H Pilz, Ralf Henkelmann, Gian M Salzmann, Norbert P Südkamp, Philipp Niemeyer

**Affiliations:** 1 Department of Orthopaedic Surgery, University of Freiburg Medical Center, Hugstetter Str. 55, D-79106, Freiburg, Germany

**Keywords:** Ankle, Osteoarthritis, Ankle Osteoarthritis Scoring System, Biomarker, Clinical trial, IGF-1

## Abstract

**Background:**

Reliable data about in vivo regulation of cytokines in osteochondritis dissecans (OCD) of the ankle are still missing. Disease-specific regulation patterns were hypothesized.

**Methods:**

28 patients with a mean age of 30.7 ± 14.8 years undergoing an arthroscopy of the ankle because of OCD were prospectively included in a clinical trial. Lavage fluids were analyzed by ELISA for levels of aggrecan, BMP-2, BMP-7, IGF-1, IGF-1R, bFGF, endoglin, MMP-13, and IL-1β. Additionally, clinical parameters and scores (FFI, CFSS, AOFAS) were evaluated and supplemented by the Kellgren Lawrence Score (KLS) for conventional X-rays and the Ankle Osteoarthritis Scoring System (AOSS) for MRI.

**Results:**

Grading of OCD lesions statistically significant increased with age and was higher in case of previously performed operations (p < 0.03). A worse clinical function reflected by low AOFAS and CFSS scores or high FFI was associated with high grading of cartilage damage or OCD (p < 0.03). Similarly, high radiological scores (KLS and AOSS) indicating progress of OA positively correlated with grading of cartilage damage and OCD. The concordance between the MRI and arthroscopic classification was overall moderate (κ = 0.52). Biochemically, only IGF/IGF-1R levels were consistently negatively associated with OCD grading, ICRS score, FFI and KLS (p < 0.05). Correlation data is supported by post hoc statistics.

**Conclusions:**

Radiological and clinical parameters in association with synovial IGF-1/IGF-1R levels indicated an increasing joint degeneration with rising OCD stage.

**Trial registration:**

German Clinical Trials Register DRKS00000365, 11/03/2008.

## Background

Although the ankle is one of the most biomechanically stressed joints, the pathophysiological understanding of associated osteochondral disorders is limited. Although generally a lot of details are known about cartilage metabolism including the effect of significant mediators and the role of biomarkers [1], reliable data about *in vivo* regulation of natural cartilage repair and biochemical consequences of osteochondritis dissecans (OCD) in the ankle is still missing. Since most of the information about controlling mechanisms in cartilage metabolism is based on studies using chondrocytes of different origins the aspect of joint specific regulatory patterns needs to be kept in mind. Several studies have shown that it is not possible to simply transfer the knowledge gained during investigations with knees to other joints as the ankle because there are significant differences in biomechanics, joint symmetry and functional reaction of chondrocytes [2,3]. It has been shown that typical arthroscopic findings or clinical symptoms are associated with disease specific radiographic changes in the course of OCD and osteoarthritis (OA) [4,5]. This study aimed to connect clinical and biochemical aspects of OCD using a re-translational approach with measurement of intraarticular cytokine levels in the course of arthroscopic surgery for OCD. In order to cover a significant spectrum of different cytokines intraarticular concentrations of bone morphogenetic protein (BMP)-2, BMP-7, endoglin (part of the BMP-receptor (BMPR)-1A complex), basic fibroblast growth factor (bFGF or FGF-2) and insulin-like growth factor (IGF-1) and its receptor as marker of intrinsic cartilage repair, interleukin (IL)-1β and matrix metalloproteinase (MMP-13) as a marker of inflammation, aggrecan as an integral part of the extracellular matrix (ECM), and the total protein content were determined. BMP-2, BMP-7, and BMPR-1A were expressed in cartilage and synovia of human knees with focal cartilage lesions [6]. BMP-2 further seemed to play an important role in surgically induced cartilage repair, because synovial expression correlated with the clinical outcome [7]. BMPR-1A was associated with the development of OA. This was also shown in patients with OCD [8], in which BMPR-1A concentrations were lower in repair cartilage covering the osteochondral defects compared to normal cartilage. The effects of bFGF on chondrocyte proliferation and differentiation are controversial, what leads to the conclusion that bFGF is necessary for the balance of reparative chondrocyte proliferation and differentiation [9]. bFGF has been shown to inhibit the anabolic effect of IGF-1 [10], a cytokine with immanent importance as a promoter of growth and matrix synthesis by chondrocytes in healthy articular cartilage. Both proteins showed increased synovial concentrations in knees with cartilage lesions [11]. Certainly the measured proteins are not the only potential markers, correlating biochemical changes following OCD with joint degeneration. But in order to focus on cytokines that characterize partial aspects of OA progress the selection was limited and other candidates as collagen fragments [12] omitted.

Besides changes of the intraarticular milieu progress of OCD and joint degeneration may be evaluated by different clinical factors. E.g. duration of complains, previous operations, characterization of associated cartilage lesions by size and depth have been described as reliable parameters [13]. These data are supplemented by different clinical scores providing a summary of region specific function [14,15]. OCD related changes are also reflected by different imaging techniques. Therefore, semiquantitative radiographic scores evaluating changes in conventional X-rays and MRI were included in the analysis. Besides the established Kellgren Lawrence Score (KLS) [16], the Ankle Osteoarthritis Scoring System (AOSS) describing and scoring the typical pathological changes in MRI was used [17].

The purpose of our study was to quantify the amount of potentially chondrodestructive and chondroprotective cytokines present in the ankle during OCD development hypothesizing stage dependent and disease specific regulation patterns. Therefore, cytokine profiles were correlated with the amount of cartilage destruction noted radiologically, arthroscopically and by determination of the clinical ankle function.

## Methods

### Study design

The study was approved by the Ethical board of the University of Freiburg (AN-EK-FRBRG-335/08) and registered at the German Clinical Trials Register (CORRCYT, DRKS00000365). A written informed consent for participation in the study was obtained from participants or, where participants are children, a parent or guardian.

As a subgroup analysis of a previously reported cohort of patients undergoing an arthroscopy of the ankle, 28 individuals with OCD of the talus were enrolled in a prospective clinical trial between November 2009 and May 2011 [17]. Patients were included in case of fulfilment of the following criteria: performance of an arthroscopy of the ankle, agreement to participate in the study by patients and/or parents in case of patients <18 years, age >10 years and <65 years. Exclusion criteria were alcohol or drug abuse, mental retardation with incapability to complete the necessary self-reports, infection. Diagnosis of OCD was confirmed by two radiologists and two orthopaedic surgeons based on criteria previously described [18].

### Specimen collection

Synovial lavage fluids of ankles of patients undergoing an arthroscopy were intraoperatively collected. Before starting the arthroscopy, 20 ml of sterile ringer solution was instilled into the joint cavity. The fluid was mixed within the joint by repeated passive flexion-extension and repeated manipulation of the posterior and anterior ankle regions, and then was aspirated as described before [11]. The aspirated volume reproducibly ranged between 8 and 13 ml. Specimens were centrifuged in order to separate the cells and then stored frozen at -80°C until analyzed [17].

### Method validation

In order to validate the method of cytokine determination in joint flushes, total protein levels of seven patients -not included in this study- were determined in effusions obtained by direct puncture and in lavage fluids obtained as described above. Statistical significance could be shown for the difference of diluted and absolute total protein concentrations as well as for the correlation of diluted and absolute concentrations (p < 0.0001). The same statistically significant differences and associations were found for bFGF; the absolute concentration of bFGF in the effusions could be calculated by multiplication with the relation of absolute (effusion) and relative (lavage) total protein content. Since the same method for lavaging the ankle joints was applied in the presented study, the same correction factor was assumed and absolute concentrations of cytokines were compared. Because the conclusions for comparison of cytokine relations are different from the comparison of absolute concentrations, both calculations were done and shown.

### ELISAs for BMP-2, BMP-7, Endoglin, bFGF, IGF-1, IGF-1R, IL-1β, MMP-13, aggrecan, BCA (bicinchoninic acid) protein assay

In order to measure concentrations of the indicated proteins, commercially available ELISA (Enzyme-linked Immunosorbent Assay) kits provided by R & D Systems (Wiesbaden-Nordenstadt, Germany) for BMP-2 (detection level (DL) = 29 pg/ml), BMP-7 (7.83 pg/ml), Endoglin (DL = 30 pg/ml), bFGF (DL = 3 pg/ml), IGF-1 (56 pg/ml), IGF-1R (DL = 156 pg/ml), IL-1β (DL = 0.125 pg/ml) and BioSource (BioSource Deutschland GmbH, Solingen, Germany) for aggrecan (DL = 1 ng/ml) were used according to the manufacturers’ instructions. Briefly, the assay employs the quantitative sandwich enzyme immunoassay technique. A specific MAb was pre-coated onto a microplate. Supernatants were applied to the wells and, after washing, an HRP-conjugated specific Ab was added to the wells. Following the next wash, colour development was proportional to protein concentration and was calculated by comparison with a standard. A colorimetric method was used in order to quantify total protein amount in the lavage fluids. The bicinchoninic acid (BCA) assay was available in kit form from Pierce (Rockford, Ill., USA) and was used according to the manufacturers’ instructions.

### Assessment of radiographic scores

#### Ankle Osteoarthritis Scoring System (AOSS)

In order to quantify the OA related changes in the ankles by MRI, the AOSS was used as previously described [17]. The description of the score composition is summarized in Table 1.

**Table 1:** **Description of the Ankle Osteoarthritis Scoring System** (**AOSS**)

**Items**	**Evaluation**	**Explanation**
**Major criteria**		
1. Depth of cartilage damage	Total points: 0–3 grade 0 (0 points): no grade 1 (1 point): <50% of total cartilage depth grade 2 (2 points): >50% grade 3 (3 points): full thickness cartilage defects	The depth of cartilage loss was qualitatively rated in relation to the height of the adjacent intact cartilage or the expected, normal cartilage contour. In doubt, the sagittal T2-weighted sequence was used for the final decision.
2. Defect of the subchondral bone	Total points: 0–3 grade 0 (0 points): no grade 1 (1 point): minimal (<2 mm) grade 2 (2 points): moderate (2-5 mm) grade 3 (3 points): severe (>5 mm)	The depth of the osseous component of the osteochondral defect was scored by estimating the distance between the actual osteochondral defect and the extrapolated subchondral cortex mainly based on evaluation of the coronary or sagittal T1-weighted sequences.
3. Osteophytes	Total points: 0–3 grade 0 (0 points): no grade 1 (1 point): minimal (<3 mm) grade 2 (2 points): moderate (3-5 mm) grade 3 (3 points): severe (>5 mm)	Size was measured from the base to the tip of the osteophyte, baseline was defined as the natural course of the bone.
4. Subchondral cysts (largest diameter)	Total points: 0–3 grade 0 (0 points): no grade 1 (1 point): minimal (<3 mm) grade 2 (2 points): moderate (3-5 mm) grade 3 (3 points): severe (>5 mm)	Subchondral cysts were defined as structures of high signal intensity on T2-weighted images in the cancellous bone underlying the joint cartilage.
5. Bone marrow edema (largest diameter):	Total points: 0–3 grade 0 (0 points) no, grade 1 (1 point): minimal (<5 mm) grade 2 (2 points): moderate (5-20 mm) grade 3 (3 points): severe (>20 mm)	Bone marrow edema was assessed as an area of increased signal intensity on T2-weighted images in the subchondral cancellous bone.
**Minor criteria**		
6. Anterolateral or anteromedial meniscoid	Total points: 0–1 0 points: no 1 point: yes	MR images were assessed for appearance of pathological anterolateral or anteromedial soft tissue structures.
7. Effusion	Total points: 0–1 0 points: no 1 point: yes	If more than a small, physiological sliver of synovial fluid was observed in the T2 images, joint effusion was assumed to be present.
8. Loose joint bodies	Total points: 0–1 0 points: no 1 point: yes	
9. Synovitis	Total points: 0–1 0 points: no 1 point: yes	Synovitis was evaluated on sagittal images and was reflected by thickening and irregularity of the normally pencil-thin rim of high signal intensity synovium.
10. Soft tissue cysts (Baker cyst analog):	Total points: 0–1 0 points: no 1 point: yes	These structures may be considered as excrescences originating from the joint capsule. They are depicted as a circumscribed mass with intermediate signal intensity on proton density-weighted and high signal intensity on T2-weighted sequences and are usually observed in the triangle of calcaneus and Achilles tendon.
**Summary**	**Total points: 0-20**	

There are 5 major (1–5) and 5 minor (6–10) criteria. The major criteria are evaluated with up to 3 points, the minor criteria with up to 1 point, respectively. The range of the total score is from 0 to 20 points. The evaluation of scores was done by two different orthopedic surgeons dedicated to knee- und ankle surgery. Both observers were masked to the patients’ biometrical data, and were trained using the scoring form. Validation of the score has been previously described [17].

#### Kellgren Lawrence Score (KLS)

This score has been assessed as described before [16] using an anterio-posterior and a lateral view of plain radiographs of the ankle. Mode of evaluation was the same as described for the AOSS. Validation of the score has been previously described [17].

#### Evaluation of clinical scores

The following clinical scores describing the function of foot and ankle were evaluated within 14 days before the operation in order to quantify a possible loss of performance. The foot function index (FFI) was introduced by Budiman-Mak et al. [19] and used in the validated german version published by Naal et al. [20]. The calcaneal fractures scoring system according to Kerr (CFSS) was originally published in order to evaluate the function following calcaneal fractures [14]. Since then, it was used in multiple settings describing function of foot and ankle. Furthermore, the ankle-hindfoot scale (AOFAS), one of the most used scores evaluating the function of foot and ankle with special regard to the lower and upper ankle joint, was used [15]. Both last scores were applied using the translated german and validated version [21]. In order to provide comparability with other studies, three different and region-specific scores were evaluated. ICRS Score for grading of cartilage damage was determined as previously described [11] during arthroscopy by the surgeon.

### Statistics

All values were expressed as mean ± standard deviation if not otherwise indicated. Correlations were determined by calculating the Spearmen coefficient (ρ) for the predominantly not normally distributed values. A cluster analysis was used to reasonably distribute the values in different groups. Based on the different clusters *post hoc* statistics (Kruskal-Wallis H-test) were used to analyze statistical significances between the grouped cytokine levels. Individual group means of scores were compared with the rank sum U-test. Statistical significance was defined when P < 0.05.

## Results

### Characterization of patients

The average age was 30.7 ± 14.8 years, the ratio male/female was 16/12 (57%/43%). 7 patients were operatively treated before (25%). 7 patients (25%) were smoker, 21 non-smoker (75%).The average body mass index (BMI) was 25.1 ± 5.4. In 20 patients a cartilage lesion graded III or IV according to ICRS was found reaching an average size of 0.69 ± 0.75 cm^2^. Duration of symptoms before operation was 25.5 ± 26.3 months. The mean KLS was 1.0 ± 0.77 (n = 28), the mean AOSS 9.7 ± 2.4, both reflecting a state of mild OCD related OA. Clinical status was evaluated using FFI (40.3 ± 25.6), CFSS (65.1 ± 22.9) and AOFAS (69.1 ± 17.4), whereas all scores indicated impaired function.

### Classification of osteochondritis dissecans (OCD)

For reasons of validation, all available standardized preoperative MRIs were independently rated by two radiologists for OCD classification. All patients underwent arthroscopy after an average of 11 weeks; assessment of OCD classification according to Berndt and Harty (modified according to Bruns) [22] in MRI and arthroscopy was compared. In 48% of all cases a difference between radiological and clinical evaluations was found. Radiological scoring was lower in five and higher in seven lesions compared to arthroscopic findings. The concordance between the MRI and arthroscopic classification was overall moderate (κ = 0.52). When looking at grade II and III cases reflecting the highest clinical importance discriminating intact or disturbed cartilage surface the concordance was only fair with a κ of 0.36.

### Association of clinical parameters with characteristics of OCD and cartilage lesions

If previous operations were performed, the ICRS grading of the cartilage damage and the grading of the OCD lesion were higher indicating a more osteoarthritic altered joint (Table 2). Duration of complains and body mass index (BMI) did not correlate with defect characteristics or OCD grading. Age was positively associated with OCD grading. A worse clinical function reflected by low AOFAS or CFSS or high FFI scores was associated with high grading of cartilage damage or OCD. Similarly, high radiological scores indicating progress of OA positively correlated with grading of cartilage damage and OCD.

**Table 2:** Overview about the intraarticular protein concentrations

	**N total**	**Average**	**SD**	**N below detection level**
MMP-13 [pg/ml]	28	12.61	28.71	22
IL-1β [pg/ml]	28	9.24	5.31	1
bFGF [pg/ml]	28	50.97	153.9	12
BMP-2 [pg/ml]	28	99.77	83.65	7
BMP-7 [pg/ml]	28	40.98	47.88	1
IGF-1 [pg/ml]	28	318.76	187.04	3
IGF-1R [pg/ml]	28	360.64	459.70	7
Endoglin [pg/ml]	28	1164.3	697.49	0
Aggrecan [pg/ml]	28	28786	37474	0
TPC [mg/ml]	28	403.30	350.96	0

SD – standard deviation, MMP-13 - matrix metalloproteinase-13, IL-1β – interleukin-1β, bFGF – basic fibroblast growth factor, BMP-2 and BMP-7 - bone morphogenetic protein-2 and 7, IGF-1 - insulin-like growth factor 1, IGF-1R - insulin-like growth factor 1 receptor, TPC – total protein content.

### Biochemical analysis

**Figure 1:**
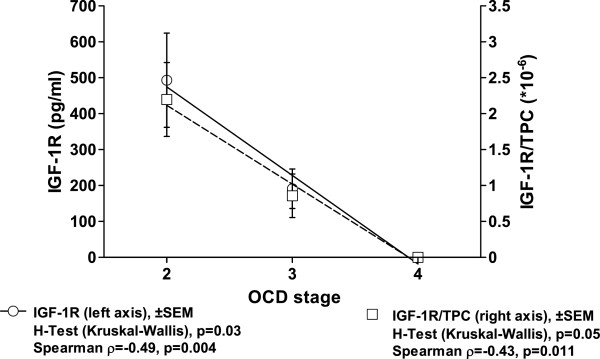
**Decreasing intraarticular levels of IGF-1R are observed with progress of oa in the ankle measured by grading of ocd according to berndt and harty.** results of the spearman correlation are indicated for absolute IGF-1R levels and concentrations in relation to the total protein content (tpc) on the bottom. the curve fit shows the results of a regression analysis (r^2^ = 0.99), which was confirmed by the Kruskal-Wallis H-test.

**Figure 2:**
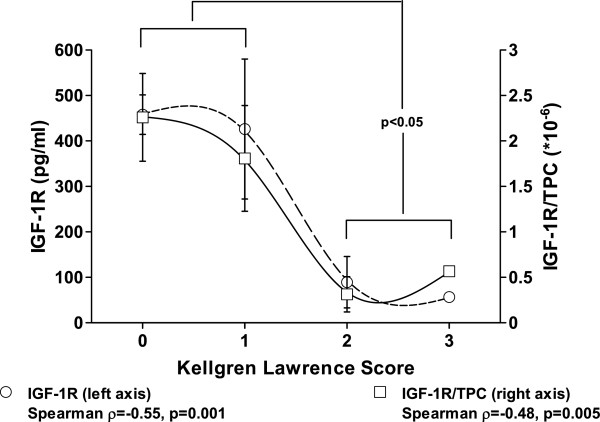
**Decreasing intraarticular levels of IGF-1R are observed with progress of OA measured by radiological changes (Kellgren Lawrence Score) of the ankle in patients with OCD.** Results of the Spearman correlation are indicated for absolute IGF-1R levels and concentrations in relation to the total protein content (TPC) on the bottom. The S-shaped association was visualized using a smoothing spline regression curve. By combining the data of KLS 0–1 and 2–3, the Kruskal-Wallis H-Test confirmed the statistically significant difference (p = 0.032).

**Figure 3:**
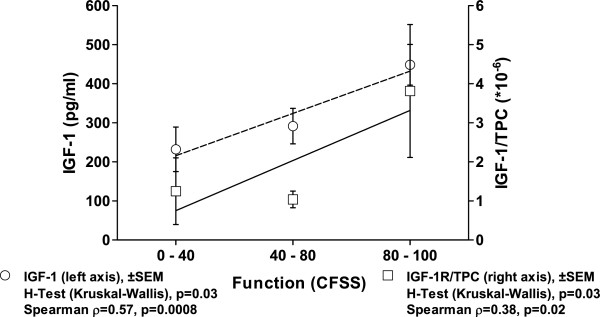
**Decreasing intraarticular levels of IGF-1 are observed with progress of OA measured by a functional score of the ankle (CFSS).** Results of the linear regression analysis (R^2^ = 0.97 and 0.83, respectively), the Spearman correlation and the *post hoc* statistics (Kruskal-Wallis H-Test) are indicated for absolute IGF-1 levels and concentrations in relation to the total protein content (TPC) on the bottom.

28 patients were included in this analysis with primarily treated OCD. Absolute concentrations found for the analyzed proteins are listed in Table 2. For correlation analysis, cytokine levels were used as absolute concentrations and concentrations in relation to TPC in order to minimize a possible dilution bias. IGF-1R levels were negatively associated with OCD grading, ICRS score, FFI and KLS (p < 0.05, ρ < -0.33, Table 3). This is confirmed by the graphical description of the association of IGF-1R concentrations and OCD grading (Figure 1) and the *post hoc* statistics (Kruskal-Wallis H-Test) confirming this statistical association (p = 0.03). Progress of OCD is usually accompanied by an increase of osteoarthritic changes in conventional X-ray measured by KLS, which has been described by our data too. Again, intraarticular IGF-1R levels were not only negatively associated with OCD stage but also with KLS (p = 0.001, ρ = -0.55, R^2^ = 0.86). The S-shaped association was visualized using a smoothing spline regression curve. This decisive association of IGF-1R and KLS was further supported by the Kruskal-Wallis H-Test (2 classes, p = 0.032, Figure 2). The importance of the IGF-1/IGF-1R system in OCD could also be demonstrated by looking at IGF-1 and the clinical function either evaluated by FFI (Table 4) or CFSS (p < 0.05, Figure 3). MRI changes in this subgroup mirrored by the AOSS demonstrated significant associations to OCD grading (p = 0.02, ρ = 0.48), ICRS score (p = 0.02, ρ = 0.40), defect size (p = 0.049, ρ = 0.41), age (p = 0.018, ρ = 0.42) and KLS (p < 0.0001, ρ = 0.68). Again, there was a negative correlation of IGF-1R and AOSS (p = 0.001, ρ = -0.62, R^2^ = 0.83). Furthermore, positive correlations to other markers of cartilage metabolism as IL-1β and endoglin (p = 0.02) emphasized the importance for IGF-1R in OCD related joint changes. There were no statistically significant correlations between age and absolute or relative synovial expression of IGF-1 (ρ = -0.24, ρ = -0.18) or age and absolute or relative synovial expression of IGF-1R (ρ = -0.13, ρ = -0.17).

**Table 3:** Properties of OCD lesions and epidemiological data

	**ICRS Score**	**Defect Size**	**OCD grading**
Previous operation^*^			
Corr.-coefficient	0.3804	0.3042	0.3789
Valid cases	28	20	28
Significance (P)	0.0229	n.s.	0.0234
**Duration of complains**			
Corr.-coefficient	0.0773	-0.0992	0.2444
Valid cases	28	20	28
Significance (P)	n.s.	n.s.	n.s.
**Age**			
Corr.-coefficient	0.3039	0.1256	0.4018
Valid cases	28	20	28
Significance (P)	n.s.	n.s.	0.0170
**BMI**			
Corr.-coefficient	0.1547	-0.2090	0.2778
Valid cases	28	20	28
Significance (P)	n.s.	n.s.	n.s.
**FFI**			
Corr.-coefficient	0.4669	0.2016	0.5232
Valid cases	28	20	28
Significance (P)	0.0061	n.s.	0.0021
**CFSS**			
Corr.-coefficient	-0.3626	-0.1845	-0.4936
Valid cases	28	20	28
Significance (P)	0.0289	n.s.	0.0038
**AOFAS**			
Corr.-coefficient	-0.2734	0.0065	-0.4588
Valid cases	28	20	28
Significance (P)	n.s.	n.s.	0.0070
**KLS**			
Corr.-coefficient	0.3954	-0.0256	0.5119
Valid cases	28	20	28
Significance (P)	0.0186	n.s.	0.0027
**AOSS**			
Corr.-coefficient	0.4035	0.4125	0.3846
Valid cases	25	17	25
Significance (P)	0.0227	0.0499	0.0288

Correlation of items characterizing an OCD lesion with regard to the accompanying cartilage lesion in the ankle with epidemiological data, function and radiological parameters. ICRS Score characterizes degree of cartilage damage. Defect size was only reported for type 3 or 4 lesions (full thickness defects). OCD grading is reported according to Berndt and Harty; BMI: body mass index, FFI: foot function index, CFSS: calcaneal fractures scoring system according to Kerr, AOFAS: ankle-hindfoot scale, KLS: Kellgren Lawrence Score, AOSS: ankle osteoarthritis scoring system, corr.-coefficient: correlation coefficient Spearman’s ρ, *U-test.

**Table 4:** Protein levels and OCD stage

	**OCD grading**	**FFI (reverse)**	**CFSS**	**AOFAS**	**KLS**	**AOSS**
**MMP**-**13/MMP-13/TPC**						
Correlation	-/-	-/-	-/-	-/-	-/-	-/-
Valid cases	28	28	28	28	28	25
Signif. (P)	n.s./n.s.	n.s./n.s.	n.s./n.s.	n.s./n.s.	n.s./n.s.	n.s./n.s.
**IL-1β/IL-1β/TPC**						
Correlation	neg. / -	- / -	- / -	-/-	-/-	-/-
Valid cases	28	28	28	28	28	25
Signif. (P)	0.040/n.s.	n.s./n.s.	n.s./n.s.	n.s./n.s.	n.s./n.s.	n.s./n.s.
**bFGF/bFGF/TPC**						
Correlation	-/-	-/-	-/-	-/-	-/pos.	-/-
Valid cases	28	28	28	28	28	25
Signif. (P)	n.s./n.s.	n.s./n.s.	n.s./n.s.	n.s./n.s.	n.s./0.050	n.s./n.s.
**BMP-2/BMP-2/TPC**						
Correlation	-/-	-/-	-/-	-/-	-/-	-/-
Valid cases	28	28	28	28	28	25
Signif. (P)	n.s./n.s.	n.s./n.s.	n.s./n.s.	n.s./n.s.	n.s./n.s.	n.s./n.s.
**BMP-7/BMP-7/TPC**						
Correlation	-/-	-/-	-/-	-/-	pos./-	-/-
Valid cases	28	28	28	28	28	25
Signif. (P)	n.s. / n.s.	n.s. / n.s.	n.s. / n.s.	n.s./n.s.	0.050/n.s.	n.s./n.s.
**Endoglin/Endoglin/TPC**						
Correlation	-/-	neg./-	pos./-	-/-	-/-	-/-
Valid cases	28	28	28	28	28	25
Signif. (P)	n.s./n.s.	0.038/n.s.	0.015/-	n.s./n.s.	n.s./n.s.	n.s./n.s.
**IGF-1/IGF-1/TPC**						
Correlation	neg./-	neg./-	pos./pos.	pos./-	-/-	-/-
Valid cases	28	28	28	28	28	25
Signif. (P)	0.018/n.s.	0.032/n.s.	0.001 / 0.021	0.030/n.s.	n.s./n.s.	n.s./n.s.
**IGF-1R/IGF-1R/TPC**						
Correlation	neg./neg.	neg./-	-/-	-/-	neg./neg.	neg./-
Valid cases	28	28	28	28	28	25
Signif. (P)	0.004/0.012	0.045/n.s.	n.s./n.s.	n.s./n.s.	0.001 / 0.005	0.006/n.s.
**Aggrecan/Aggrecan/TPC**						
Correlation	-/-	-/-	-/-	-/-	-/-	-/pos.
Valid cases	28	28	28	28	28	25
Signif. (P)	n.s./n.s.	n.s./n.s.	n.s./n.s.	n.s./n.s.	n.s./n.s.	n.s./0.048

Correlation of intraarticular protein levels of patients with OCD with OCD stage according to Berndt and Harty, functional scores and OA-related changes in conventional X-ray or MRI, signif. (P): p-value for statistical significance Spearman’s ρ, FFI: foot function index, CFSS: calcaneal fractures scoring system according to Kerr, AOFAS: ankle-hindfoot scale, KLS: Kellgren Lawrence Score, AOSS: ankle osteoarthritis scoring system, TPC: total protein content. Statistical tests refer to correlations to the absolute protein concentrations and the protein concentrations in relation to TPC.

## Discussion

The presented data confirm typical correlations between stage of OCD and radiographic changes, clinical function, and other indicating factors for OA as age or degree and size of cartilage damage. The main finding of the study is the association of synovial IGF-1/IGF-1R levels with OCD stage. Decreasing concentrations of these indicator proteins signal increasing joint degeneration evaluated by radiographic scores and deteriorating clinical function.

IGF-1 enhances aggrecan synthesis by articular cartilage cells or explants, which has been demonstrated in cell culture experiments and using in vivo animal models [23]. IGF-1 is also produced by chondrocytes and stored within the extracellular matrix of cartilage, probably bound to proteoglycans, particularly to the cell-surface located syndecans and the IGF-1 binding proteins [24,25]. Although IGF-1/IGF-1R have not yet been described with specific alterations of joints following OCD, enhanced IGF-1 secretion was demonstrated in knees with circumscribed cartilage lesions [11], confirming the importance of this protein for cartilage metabolism. According to our results, IGF-1 and IGF-1R were the only synovial proteins showing a consistent association with disease progress, because both were progressively diminished with advance of OCD. Limiting has to be stated that the data presented are only of a single time point in a cross-sectional study, making an extrapolation for determination of OCD progress difficult. As known, age plays a decisive role in OCD development and is important for prognosis. The data for relations of IGF-1 expression and age are contradictory, showing either positive [26] or negative [27] associations probably depending on the sample population and the compartment, where the specimens were collected. In this study no significant correlation could be found for synovial IGF-1/IGF-1R expression and age, neither in the subpopulation with OCD nor in whole population included [17]. During OCD, degeneration of cartilage with change to a more fibroblastic, cell-rich phenotype is caused by the sclerosis of the subchondral bone [8]. This seems to be associated with diminished IGF-1/IGF-1R secretion. The IGF-1 receptor is a transmembrane protein transmitting the downstream signaling by insulin receptor substrate-1 (IRS-1) that is functionally modified by extracellular signal-regulated kinases (ERK) [28]. The intraarticularly measured receptor is probably a shed part, which potentially modifies IGF-1 binding capacity to the intact IGF-1R.

It previously could be shown by an immunohistological analysis in the knee that there is not only a difference in phenotypic appearance of cartilage in OCD but also in biochemistry, because expression of BMPR-1A was decreased in the de-differentiated repair tissue [8]. In contrast, for synovial concentrations of BMP-2, BMP-7 or endoglin (CD105) a correlation to OCD grading in the ankle could not be demonstrated. The results of this study only describe a summary effect of the reaction of the whole joint, but do not allow to discriminate which cell population is responsible for the phenomenon of reduced IGF-1 secretion with progress of OCD. Previously, we described an association of pain in circumscribed cartilage lesions and synovial IGF-1 expression in the knee [26]. Although the evaluated clinical scores partially consider pain perception this issue was not particularly addressed in this study. Furthermore, data found in clinical studies in the knee may not easily be transferred to the ankle, because biomechanics differ in terms of load, symmetry [29,30], chondrocyte function and biochemical reactivity [2]. Whether the observed regulation is a specific cartilage problem or may be related to metabolic disorders of glucose balance [26] was also not addressed in this analysis. Although a lot of publications relate IGF-1 and its receptor to natural or pathological cartilage turnover [11], this is the first study suggesting a special role for this protein in OCD development. Operations, progressive OA and other catabolic situations are associated with at least temporarily elevated levels of IL-1β, however, this could not be found in the presented study. This may be explained by the fact that there was a significant period of time between onset of symptoms and operation, during which the lavage was collected. Considering this and the overall low mean radiographic OA scores, all of the recruited patients of this study may be considered to be in a chronic state of mainly mild OCD related OA. Similarly, in a study looking for TNFα levels in knees with OA a correlation to KLS was missing [31]. OA development in the ankle may be caused by a primarily disturbed biomechanics as seen after fractures or impingement syndromes. Data presented suggest a primarily and predominantly disturbed biochemistry in OCD with differential regulation of IGF-1/IGF-1R, which is possibly more specific as the disturbed biochemistry in hemophilia, in which the whole inflammatory cascade is activated driven by the neutrophil influx. This would possibly offer a defined and specific pharmaceutical way for OA prevention in OCD by IGF-1 substitution. A combination with stage adapted surgical treatment approaches as previously summarized [32] would also be a possible proposal in order to increase chances for treatment success.

Limitations of the study are the number of included patients and the lack of a possibility to attribute the observed reaction to a certain cell or tissue type. Since this study has cross-sectional character, it is not possible to determine a definite cause and effect relationship and there is no specific control lavage fluid. Despite this fact, clinical studies will be necessary in future, because it is difficult to transfer this clinical situation into an animal model. Furthermore, on the basis of the presented data it is not possible to clarify, whether diminished IGF-1/IGF-1R levels are a symptom of OCD or a reason for the onset.

## Conclusions

Summarizing, on the basis of valid clinical and radiological data we were able to identify IGF-1 and IGF-1R as markers of OCD development in the ankle, both decreasing with OCD progress.

## Competing interests

All authors declare that they have no financial and personal relationships with other people or organizations that could potentially and inappropriately influence (bias) their work and conclusions.

## Authors’ contributions

HS (hagen.schmal@freenet.de) was responsible for the conception and the design of the study, for obtaining of funding, the analysis and interpretation of the data, tutorial of Mr. Henkelmann, drafting of the article, and the final approval of the submitted article. I H P (ingo.hinrich.pilz@uniklinik-freiburg.de) supported the lab work, supervised the sample analysis, was responsible for sample storage, revised the article draft critically, and approved the final version of the article. R H (ralfhenkelmann@googlemail.com) was responsible for the collection, assembly and management of data, performed the ELISAs, contributed to the article draft, calculated the scores and the descriptive statistics, and approved the final version of the article. G M. S (giansalzmann@yahoo.com) performed operations und by this provided study material and acquired patients, contributed to data management with his statistical expertise, and approved the final article. N P. S (norbert.suedkamp@uniklinik-freiburg.de) was involved in the conception and design of the study and the trial protocol, provided study material, contributed to obtainment of funding, gave administrative support, critically revised the article draft, and approved the final version of the article. P N (PhNiemeyer@gmail.com) was involved in the conception and the design of the study and the trial protocol and gave significant input in the fundamental considerations regarding OCD and its treatment, contributed to the obtainment of funding, critically revised the article draft, and approved the final version of the article. All authors take responsibility for the integrity of the work as a whole, from inception to finished article.

## Pre-publication history

The pre-publication history for this paper can be accessed here:

http://www.biomedcentral.com/1471-2474/15/169/prepub
